# State-of-the-Art, and Perspectives of, Silver/Plasma Polymer Antibacterial Nanocomposites

**DOI:** 10.3390/antibiotics7030078

**Published:** 2018-08-17

**Authors:** Jiří Kratochvíl, Anna Kuzminova, Ondřej Kylián

**Affiliations:** Department of Macromolecular, Faculty of Mathematics and Physics, Physics Charles University, Prague 18000, Czech Republic; kratji@seznam.cz (J.K.); annakuzminova84@gmail.com (A.K.)

**Keywords:** non-equilibrium plasma, silver, antibacterial coatings, plasma polymers, nanocomposites

## Abstract

Urgent need for innovative and effective antibacterial coatings in different fields seems to have triggered the development of numerous strategies for the production of such materials. As shown in this short overview, plasma based techniques arouse considerable attention that is connected with the possibility to use these techniques for the production of advanced antibacterial Ag/plasma polymer coatings with tailor-made functional properties. In addition, the plasma-based deposition is believed to be well-suited for the production of novel multi-functional or stimuli-responsive antibacterial films.

## 1. Introduction

As shown in numerous recent reviews, road-maps, and “white” papers [1,2,3,4,5,6,7], non-equilibrium plasmas may be successfully used in various fields, such as from the automotive or aerospace industry to waste and pollution control, or from the production of advanced functional biomaterials to the design of smart textiles. The tremendous boom that plasma-based technologies experienced in the last few decades is mainly given by their unique features. These comprise a relatively low-temperature nature of the processes that employ plasma, allowing processing of heat-sensitive materials, including polymers, to treat or coat virtually any material without compromising the bulk properties of treated objects or high versatility in terms of materials that may be deposited by plasma-based techniques (e.g., metals, metal-oxides, plasma polymers). In contrast to techniques that utilize chemical synthesis, plasma processes are dry with no or limited use of solvents or potentially harmful chemical substances, making them, in many cases, a highly valuable “green” alternative to “wet” chemical methods. Enormous advancement in the use of plasma technologies was also enabled by recent progress in plasma science and technology, especially in the case of atmospheric pressure discharges, as well as by close collaborations of plasma physicists with chemists, biologists, and medical doctors. This broadened the range of possible applications of non-equilibrium plasmas that nowadays include sterilization/decontamination of surfaces [8,9,10,11,12,13], plasma medicine [14,15,16,17], agriculture [18,19,20,21,22], or as it will be discussed in this review, the production of antibacterial coatings [23,24,25]. 

In order to highlight the state-of-the-art plasma-based strategies for the deposition of silver-containing antibacterial coatings, the article is organized as follows: In Section 2, different approaches that may be used for the suppression of bacteria adhesion or biofilms formation will be briefly summarized, with emphasis given to Ag-containing nanocomposites. Different plasma-based strategies that were developed for the production of Ag/plasma polymer nanocomposites will be presented in Section 3. Finally, Section 4 will cover the challenges and future perspectives of the plasma deposition of antibacterial coatings. 

## 2. Antibacterial Coatings

Preventing the bacterial colonization of surfaces is a key requirement to limit the spread of infections. Although this is important in various fields (e.g., the textile industry, food packaging production, or space missions [26,27,28,29,30]), most of the attention is devoted to surfaces used in medicine and healthcare services. Here, the presence of bacteria on medical instruments, tools, accessories etc. may have catastrophic consequences. Although many of the factors leading to bacterial infection can be easily avoided by appropriate hygiene procedures in the hospitals, it is impossible to completely avoid bacterial infections. For example, this is true in the case of medical implants. It is well known that planktonic bacteria present in body fluids may adhere to the implant surface in vivo, multiply, and start to form complex and highly resistant communities, such as biofilms, where bacteria can survive for extended periods of time. The biofilms may then act as persistent reservoirs for pathogens which may trigger infections. This, in turn, often necessitates re-operation and replacement of the infected implant that not only increases costs, but represents serious risk—especially for elderly patients. A promising strategy for reducing the occurrence of such undesirable events is to avoid the initial attachment of bacteria to the implant’s surface. Generally, this can be done by following two strategies [31]. 

The first one is based on coating implants with non-fouling thin films, i.e. films that are capable of resisting protein adsorption or bacteria adhesion. The typical examples of such materials are *poly (ethylene glycol)* (PEG) derivatives or zwitterionic polymers, that have proved to be able to reduce or inhibit bio-fouling [32,33,34,35,36]. However, it has to be stressed that in most of the in vivo experiments, surfaces led only to the delay of biofilm formation and restricted longer-term stability and performance. Furthermore, non-fouling coatings are prone to damage during their handling. 

The second way in which to combat biofilm formation is by the production of surfaces that actively kill bacteria. This may be achieved either by contact-killing, or by release-based strategies. The contact-killing of adhered bacteria is achieved either by antimicrobial compounds covalently anchoring to the material surface. Examples include quaternary ammonium compounds, cationic peptides, or enzymes [23,37,38], or the design of biomimetic nanostructured coatings with high-aspect-ratio surface topographies that exhibit biocidal efficacy [39,40]. In the case of release-based coatings, antibacterial action is connected with the gradual leaching of antibacterial agents from the coatings into the surrounding media. The antimicrobial agents may either be organic or inorganic [23,31,41]. Regardless of the antibacterial agent used, its local delivery at a specific site ensures a high local dose without exceeding the systemic toxicity. Because of this, various techniques for the production of such antibacterial coatings were developed, including plasma-based ones that utilized organic antibiotics [42,43]. 

However, despite the success of these approaches, silver-based materials have been receiving increasing attention since the 1960s. This was caused by ongoing reports on the development of the resistance of certain bacteria to commonly used antibiotics (e.g., [44,45,46,47,48,49,50]). Even though this is also the case for silver, where reports exist which indicate the occurrence of silver-resistant bacteria [51,52], the development of bacterial resistance towards Ag is highly improbable due to the multiple possible pathways that may lead to bacterial damage by silver nanoparticles (NPs). The exact mechanism of the antibacterial action of Ag NPs is still not fully understood, and is a frequent topic of discussion and controversy. Ag NPs may either induce the formation of reactive oxygen species (ROS) or may release in aqueous media, such as highly bioactive silver ions. The ROS and Ag^+^ ions produced subsequently interact with DNA or the thiol groups of molecules present in the cytoplasm, cell membrane, and inner membrane of mitochondria, that may result in changes in permeability, disturbance of respiration, leakage of intracellular content, inhibition of protein synthesis and function, and cell apoptosis or necrosis [52,53,54,55]. 

Naturally, the silver NPs and released silver ions may interact not only with bacteria, but also with tissue cells. Fortunately, the health risks associated with systemic absorption of Ag^+^ ions are rather low [56]. Nevertheless, in order to avoid any undesirable side-effects connected with silver release, its dosage has to be carefully controlled and regulated, which is one of the key challenges connected with the application of silver-based antibacterial nanocomposites. 

## 3. Antibacterial Ag/Plasma Polymer Nanocomposites

Numerous studies have been devoted to the functionalization of surfaces (mostly polymeric ones) by antibacterial nanosilver (e.g., [57,58,59,60,61,62]). However, a limitation of the proposed methods was the poor adhesion of silver to substrates, which often led to the washing-out of Ag NPs from the substrate, which came with unacceptable pollution of the environment. Although it was demonstrated by different groups that this effect may be substantially suppressed by plasma activation/functionalization of substrate materials prior to the silver deposition (e.g., [63,64,65,66,67,68]), in many cases the stability of Ag layers still remained questionable. Because of this, alternative strategies based on the embedding of Ag NPs into a supporting matrix were suggested. From these points of view, plasma polymers are considered to be highly promising matrix materials, i.e. macromolecular solids that are created when an organic vapour or precursor passes through a plasma [69,70,71,72,73,74]. In contrast to conventional polymers, plasma polymers have a random and irregular structure with higher degrees of crosslinking, and a branched structure. Despite their complex structure, plasma polymers offer certain advantages- they can be deposited in the form of conformal, pin-hole free thin films on any substrate material with well-controlled thicknesses at a nanometre scale. Furthermore, their properties that can be tuned by a wide range of operational conditions (e.g., applied power, precursor/working gas mixture, pressure) span from soft to hard polymers, from bio-fouling to non-fouling, or from films that are stable, swelling, or dissolvable in aqueous media. The use of plasma polymers thus may not only improve the fixation on Ag NPs and avoid their release to surrounding media, but, due to the high flexibility of plasma polymers in terms of their physico-chemical and bio-adhesive properties, also tailor their antibacterial or bio-fouling performance. In the following two subsections, different approaches that were tested, with the aim of producing Ag/plasma polymer nanocomposites, will be summarized. 

### 3.1. Layered Nanocomposites

**Figure 1:**
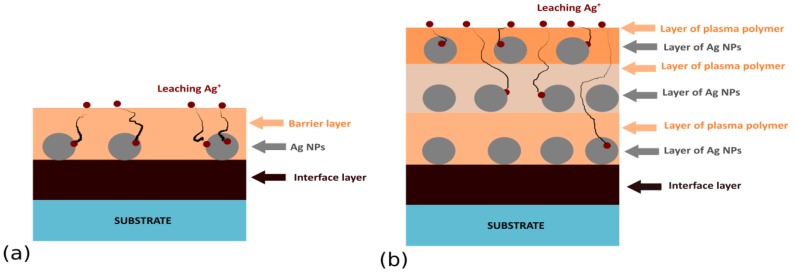
Common structure of (**a**) sandwich and (**b**) multi-layered Ag-based nanocomposites.

**Figure 2:**
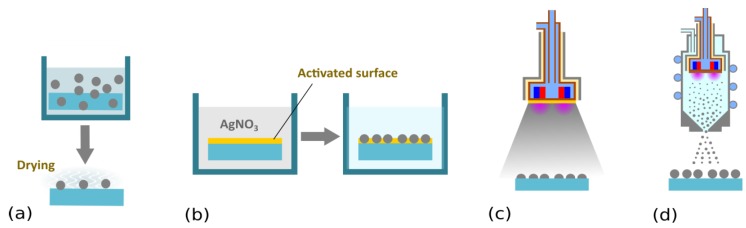
Schematic representation of possible strategies for coating substrates with Ag NPs. (**a**) Immersion into a colloidal solution of Ag NPs, followed by drying (e.g., [63,67,75]). (**b**) Salt reduction. Substrates are commonly exposed to silver nitrate solution. AgNO_3_ is, in the second step, reduced by an appropriate reducing agent (e.g., sodium citrate, sodium borohydride, or even H_2_ plasma) [76,77,78,79]. (**c**) Direct current (DC) magnetron sputtering. In this low-pressure vacuum-based plasma deposition technique, a silver target mounted onto a DC magnetron is subjected to a flux of highly energetic ions produced in the plasma bulk. This leads to the emission of silver atoms that condense on a substrate located in the deposition chamber. Depending on the deposition conditions (magnetron current, pressure, deposition time, etc.) various silver nanostructures are formed that range from individual separated nanoislands to interconnected Ag networks (e.g., [59,80,81,82,83,84,85,86,87]. (**d**) Deposition of Ag NPs by means of a gas aggregation source equipped with a silver target. Gas aggregation sources based on magnetron sputtering were introduced by Haberland et al. [88] and since then were successfully applied for the fabrication of different kinds of nanoparticles (for more information, readers should refer to the instance to monography [89] or recent review articles [90,91,92,93]). In contrast to sputter deposition, silver nanoparticles are already formed in a volume of the aggregation chamber as a result of gas-phase nucleation of sputtered atoms, which is followed by the coagulation or coalescence of growing nanoparticles. As shown by our group, the fact that NPs are formed in the gas-phase means their properties are not dependent on the substrate material, which makes it possible to independently control the size and number of deposited NPs [94,95].

Sandwich or multi-layer structures (see Figure 1) represent the first family of antibacterial Ag/plasma polymer nanocomposites. Such coatings typically consist of Ag NPs overcoated with a thin top layer of plasma polymer (typically from several nanometers to tens of nanometers) that fixes Ag NPs on a substrate material and acts as a diffusion barrier for leaching Ag^+^ ions. Ag NPs may be deposited on a substrate either by immersion into a colloidal solution of Ag NPs followed by drying, salt reduction, or physical methods, such as evaporation, magnetron sputtering, or the use of gas aggregation sources (GAS) of silver NPs (see Figure 2). The physical methods of Ag NPs deposition are highly advantageous, as they limit the possible uncontrolled aggregation of Ag NPs on surfaces, ensure high purity of silver NPs, and may be easily combined with other low-pressure deposition techniques used for plasma polymer matrix deposition. In order to facilitate the adhesion of Ag NPs onto a substrate additional bottom layer (often plasma polymer film) is used as an interface between the substrate and Ag NPs.

**Figure 3:**
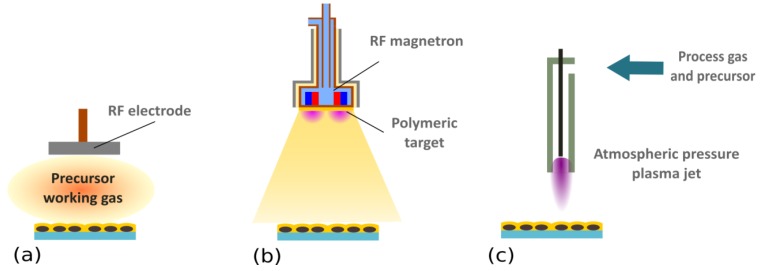
Schematic representation of possible plasma-based strategies for the overcoating of Ag NPs. (**a**) Plasma-enhanced chemical vapor deposition (PE-CVD). In this method, organic vapours or precursors are introduced into the plasma, where they are activated and fragmented. Formed radicals that condense on a substrate subsequently undergo free radical chain growth polymerization (for more details, please refer to the monographies [69,71,72] or reviews [2,24,74]). (**b**) RF magnetron sputtering of a polymeric target. In this case, starting material is supplied in the form of a solid-state polymeric target that is attached onto a magnetron. Atoms, molecules, and molecular fragments sputtered from the polymeric target consequently participate in plasma polymerisation, as in the PE-CVD. In contrast to sputtering of metallic targets, polymers are not conductive, meaning that RF power has to be applied. Magnetron sputtering was employed for the production of a wide range of plasma polymers, including C:H, C:H:N:O, or C:F ones (e.g., [103,104,105,106,107,108,109]). (**c**) Atmospheric pressure plasma jets. These systems are based on PE-CVD process, but the gaseous or liquid precursors are introduced to the plasma ignited at atmospheric pressure. Different configurations of atmospheric pressure plasma jets were demonstrated to be suitable for the deposition of various plasma polymer films (e.g., [110,111,112,113,114,115,116,117,118]).

In most cases, low pressure deposition systems (plasma-enhanced chemical vapor deposition or magnetron sputtering, see Figure 3a,b) were selected for the deposition of the base and top layers. The choice of overcoat material and its thickness strongly influenced the antibacterial action of the resulting coatings. For instance, the Kiel group, in their detailed study using Ag NPs that were sandwiched in between RF sputtered *polytetrafluoroethylene* (PTFE) and a layer of either plasma-sputtered PTFE, plasma-polymerized *hexamethyldisiloxane* (ppHMDSO), or SiO_x_ films prepared by plasma polymerization of HMDSO with admixed oxygen, proved that the release of silver ions and also the antibacterial efficacy of produced coatings strongly depended on the overcoat material [96,97], and the highest Ag^+^ release and bacteria-killing rates were observed for SiO_x_ films. Similar conclusions were drawn by Kuzminova et al. [98], who used multi-layer structures with alternating layers of ppHMDSO or SiO_x_ and Ag NPs prepared by the PE-CVD and GAS system. Better antibacterial behavior of an SiO_x_-like matrix was ascribed to the different wettability of ppHMDSO and SiO_x_—whereas ppHMDSO is hydrophobic and thus acts as a barrier for water penetration into the depth of the coatings, SiO_x_ films are hydrophilic and more water permeable. This was confirmed by Blanchard et al. [99] who studied water penetration into ppHMDSO and SiO_x_ thin films by means of neutron reflectometry. However, recent results of Kylian et al. [100] showed that even for highly hydrophobic C:F top layers (water contact angles of prepared coatings up to 165°), a strong antibacterial character of the coatings may still be observed when only a thin C:F layer (10 nm) is used. Such a result suggests that coating wettability is not the only parameter, and that the morphology of the coatings—especially the presence of crevices and defects in the overcoat layer which make Ag NPs accessible by water—has to be considered as well. In addition, the release of antibacterial Ag^+^ responsible for antibacterial action of produced coatings was found to be strongly linked with the number of silver NPs in the coatings, as well as with the thickness of the top barrier layer. This allows for the fabrication of the coatings with either a burst-release of silver ions, or a slow but temporally stable leaching of Ag^+^ ions. 

Although the low-pressure deposition systems were traditionally used for the production of plasma polymer overcoats, the possibility to also use atmospheric pressure deposition systems was suggested by Deng et al. [101,102]. These authors used an atmospheric pressure plasma jet operated in a mixture of N_2_/O_2_/*tetramethyldisiloxane* (TMDSO) to produce a barrier layer deposited on top of PET fabrics coated with Ag NPs. It was demonstrated that the antibacterial effect of such prepared fabrics on *P. aeruginosa*, *S. aureus*, *C. albicans* and *E. coli* may be varied by the thickness of the top layer. Furthermore, the top layer was confirmed to enhance the stability of antibacterial effect, and no variation of antibacterial action was observed after 10 washing cycles [101].

### 3.2. Direct Embedment of Ag NPs into Plasma Polymer Matrix

**Figure 4:**
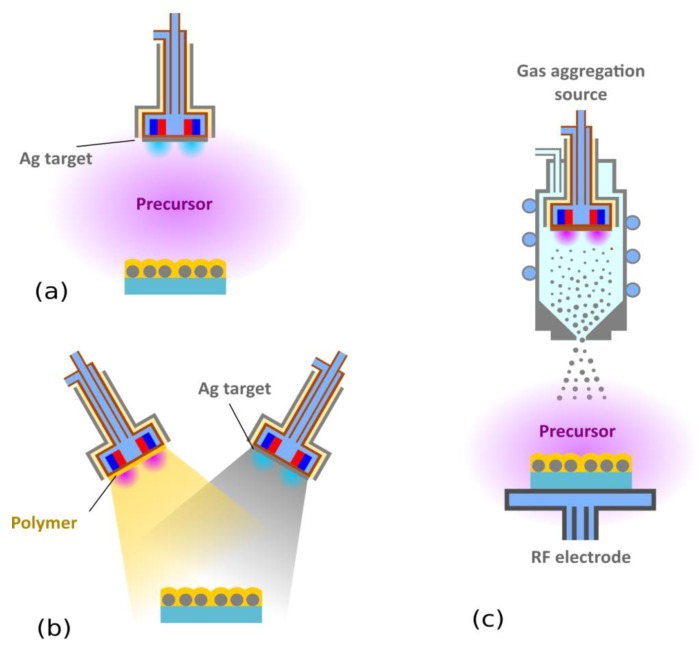
Different approaches for the production of Ag/plasma polymer nanocomposites: (**a**) Simultaneous sputtering and plasma polymerization, (**b**) deposition from two independent magnetrons, and (**c**) a combination of a gas aggregation source and plasma polymerization.

As shown in the previous section, layered silver-containing structures are highly effective at killing bacteria. However, the deposition procedure involves multiple steps, which is, from a technological point of view, a limiting factor. Consequently, alternative approaches for Ag-based nanocomposite production were developed, in which Ag nanoparticles are incorporated directly into the growing plasma polymer matrix. One of the first attempts toward this direction were reported by Favia et al. [119] and Sardella et al. [120]. These authors prepared conformal coatings with silver NPs embedded in a *polyethylene oxide*-like (PEO-like) matrix, using PE-CVD from RF glow discharges fed with Ar, and *diethylglycol-dimethyl-ether* (DEGME) with simultaneous sputtering from the Ag RF electrode in an asymmetric parallel-plate configuration (see Figure 4a). Due to the reactor asymmetry, negative DC self-bias developed on the smaller Ag electrode, which led to the bombardment of the Ag target by highly energetic ions inducing silver sputtering. Emitted Ag atoms impinged upon the plasma polymer surface, which grew simultaneously by plasma polymerization. Ag atoms, due to their limited diffusion in a cross-linked matrix, subsequently formed stable metal nanoparticles through an aggregation process. The properties of the resulting nanocomposites (Ag filling factor, size of Ag NPs, and PEO-like character of the matrix) were controlled by operational parameters (power, pressure, and Ar flow). Based on the disk diffusion test that was performed with *S. epidermidis,* it was reported that the inhibition area correlated with the Ag content in the coatings. Similar approaches using simultaneous plasma polymerization and Ag sputtering was since then employed by other research groups, which utilized different precursors/working gas mixtures. Prepared nanocomposites were tested towards both Gramm-negative and Gramm-positive bacteria (for a summary of results, please refer to Table 1). Furthermore, it was found that Ag/HMDSO nanocomposites with properly-tuned silver content may not only kill bacteria, but also drastically reduce microbial adhesion [121,122].

The great advantage of simultaneous sputtering and plasma polymerization is that it is a single-step process. However, as the sputtering and plasma polymerization are fully coupled, it is not possible to independently tailor the size of Ag NPs and their amount in the films and matrix properties. In order to overcome this limitation, another procedure that is based on co-sputtering from two independent magnetron sources may be used (see Figure 4b). The volume fraction of silver in a matrix material is, in this case, controlled by the power applied to the individual magnetrons. This approach, which is also applicable for the production of inorganic Ag containing antibacterial nanocomposites (e.g., Ag/silica [123], Ag/hydroxyapatite [124], or Ag/TiO_2_ [125]), was used for the fabrication of Ag/C:F antibacterial films by Zaporojtchenko et al. [126]. It was reported that the nanocomposites produced steadily supplied silver ions for a long period of time (reported values were for 300 days) and exhibited antibacterial effects towards *S. epidermidis*, *S. aureus,* and *P. aeruginosa*. Moreover, an addition of a small amount of Au (1%) was found to substantially increase the release rate of silver ions (by one order of magnitude) [126]. This effect is explained by the formation of galvanically-coupled Ag and Au NPs, because in the galvanic pair, silver is more active than gold, and the presence of gold enhances Ag^+^ ion formation. 

Another strategy that makes it possible to completely decouple the deposition of Ag NPs and the plasma polymer matrix is based on the use of gas aggregation sources [134,135]. This approach was recently used by Vaidulych et al. [133] for the deposition of Ag/a-C:H nanocomposites. In order to produce hard coatings, the substrates were placed on a RF electrode, used for a-C:H matrix deposition, perpendicularly to the direction of a beam of Ag NPs produced by the GAS system (Figure 4c). As it was shown in a previous study focusing on Cu/a-C:H nanocomposites [136], the volume fraction of metallic NPs in a-C:H matrix and connected antibacterial efficiency may be tuned either by the current applied onto the DC magnetron in the gas aggregation source, or by changing the duty cycle of RF power used for the matrix deposition. Furthermore, it was reported that antibacterial action of Ag/a-C:H coatings may be enhanced by the partial etching of nanocomposites by plasma treatment performed in the same deposition chamber resulting in partial removal of a carbonaceous layer from the outermost surface of produced nanocomposites, thus making Ag NPs more accessible for water [133].

**Figure 5:**
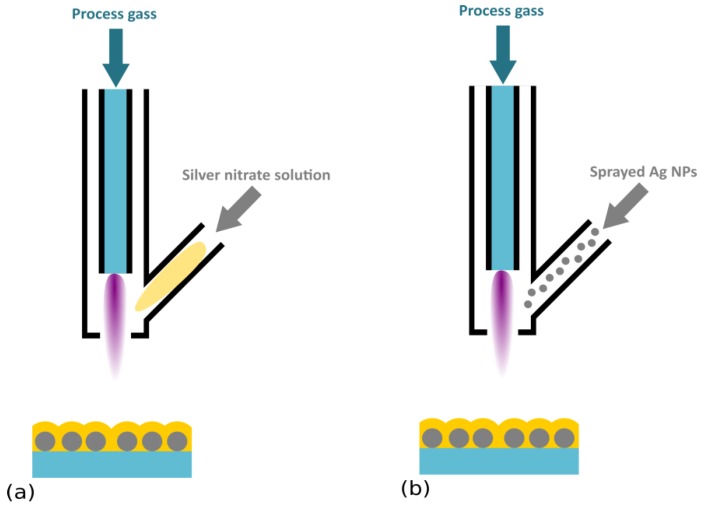
Schematic representation of atmospheric pressure plasma systems for the deposition of Ag/plasma polymer nanocomposites. (**a**) Plasma jet with injection of silver nitrate solution, and (**b**) plasma jet fed with suspension of Ag NPs.

A completely different one-step process applicable for the deposition of Ag/plasma polymer antibacterial films was proposed by Zimmerman et al. [137], Beier et al. [138], and Gerullis et al. [139]. These authors used an atmospheric pressure plasma chemical vapor deposition technique, in which a HMDSO precursor was introduced into the atmospheric pressure plasma, together with silver nitrate by a modified jet nozzle (Figure 5a). A strong antibacterial effect of nanocomposites produced in this way on *E. coli* was shown even after 10,000 washing cycles [138]. Atmospheric pressure deposition was finally also tested by Deng et al. [140], where they directly introduced Ag NPs (100 nm size) instead of silver nitrate into the feed gas (N_2_ with admixing of O_2_ and TMDSO) (Figure 5b). According to the reported results, prepared coatings exhibited strong antibacterial effects against Gramm-negative *E. coli,* and a modest effect on Gram-positive *S. aureus*. A similar approach was recently employed by Ligouri et al. [141] for the production of plasma-polymerized *polyacrylic acid* with embedded Ag NPs. Disk diffusion tests with *E. coli* confirmed the antibacterial efficacy of fabricated coatings.

## 4. Perspectives and Challenges

**Figure 6:**
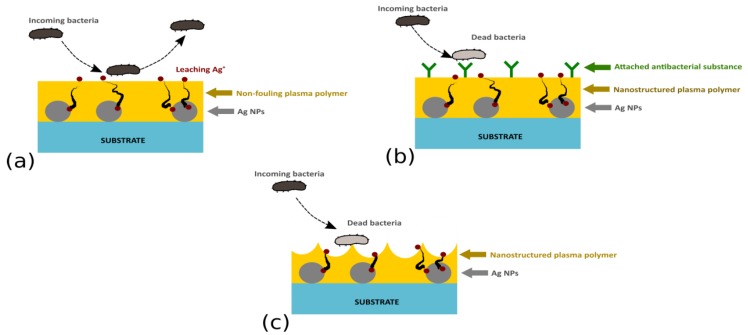
Ag-based multi-functional coatings with (**a**) non-fouling character, (**b**) Ag/plasma polymer nanocomposites with covalently immobilized antibacterial agents, or (**c**) a nanostructured plasma polymer top layer.

Plasma-based techniques have been shown to present itself as an interesting option for the production of antibacterial Ag-based coatings. In addition, plasma-based deposition technologies are well-suited for the production of novel antibacterial coatings with enhanced functionality. This refers not only to the possibility to produce coatings with a multi-approach character that may combine the antibacterial action of Ag NPs with the non-fouling nature of a polymeric matrix (e.g., PEO-like plasma polymers, Figure 6a), silver/plasma polymer nanocomposites with covalently immobilized bactericidal substances (Figure 6b), or surface nanotopography (Figure 6c), but also coatings with (i) a temporally non-monotonous release of silver ions, (ii) so-called multi-release coatings that employ two or more antibacterial agents, or even (iii) thin films with Ag^+^ release induced by an external stimuli, i.e., coatings that were suggested to facilitate prevention of bacterial infections (e.g., [31]). 

**Figure 7:**
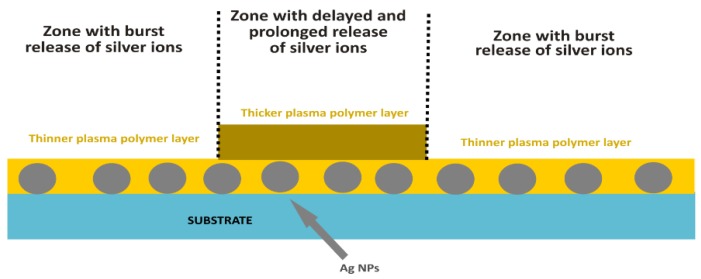
Scheme of Ag/plasma polymer coating with non-monotonous kinetics of release of silver ions.

Regarding coatings with non-monotonous kinetics in the release of silver ions, two approaches are under investigation. The first one is based on the possibility to tune Ag^+^ release by the thickness of the barrier top layer (Figure 7). In this case, different parts of Ag NPs containing films may be coated by plasma polymer films with different thicknesses. Zones with thin top layers will ensure burst release, whereas zones coated with thicker films will be characterized by the delayed and prolonged leaching of a smaller amount of Ag^+^. The same is expected to be achieved by the second approach that is based on multi-layer coatings, or the coatings with a depth gradient in the number of embedded NPs. Such materials should fulfill the requirement of long-lasting (several months of) antibacterial action needed to prevent infections on implants. 

**Figure 8:**
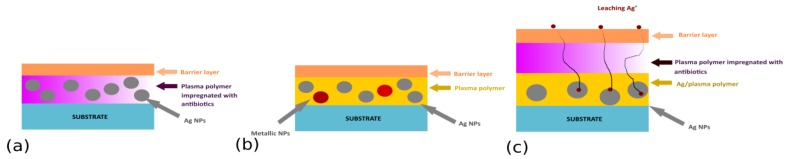
Different architectures suggested for multi-release coatings.

In the case of multi-release coatings, different antibacterial agents (e.g., silver and copper NPs or Ag NPs and antibiotics) with different release kinetics and/or bactericidal effects can be combined. Such coatings should significantly reduce the induction of bacterial resistance and guarantee synergic antibacterial action, thus enhancing antibacterial efficiency. Possible structures under consideration include silver-containing plasma polymer films impregnated with antibiotics (Figure 8a), sandwiched structures with different metallic NPs with antibacterial character (Figure 8b), or multi-layered coatings prepared by a step-by-step deposition, in which individual layers will be loaded with different antibacterial agents (Figure 8c). 

Plasma-based deposition techniques may also be utilized for producing coatings with stimuli-responsive behavior. These materials benefit from the ability of some materials to undergo volume or structural changes when exposed to a particular trigger. This property may be utilized for controlling the release of antibacterial agents, including silver ions, from the coatings “on demand”. The first example of Ag/plasma polymer nanocomposites with this ability was reported by Kulaga et al. [142]. These authors coated polypropylene surgical mesh with a layer of plasma-polymerized *maleic anhydride* (MA), impregnated with silver NPs and coated by a barrier layer (plasma polymerized MA) that blocked the spontaneous release of silver from the coatings. Tailored release of silver ions was then achieved by mechanical stimulation of the coatings that led to the formation of cracks in the barrier layer. Moreover, plasma polymerization may also be used for the production of other types of stimuli-responsive plasma polymers where changes in the temperature or pH acts as a trigger. For instance, Pan et al. [143], Spridon et al. [144], Chen et al. [145], and Moreno-Couranjou et al. [146] synthetized thermo-responsive plasma-polymerized *poly(N-isopropylacrylamide)* or *N-vinylcaprolactam* films. Muzammil et al. [147] reported on the pH-responsive coatings prepared by the plasma co-polymerization of *acrylic acid* and *octafluorocyclobutane*. Although none of the already developed stimuli-responsive plasma polymers were used for the fabrication of Ag-containing nanocomposites, this option still shows a great deal of promise. 

Finally, it has to be mentioned that despite the substantial and undisputable progress in the field of antibacterial Ag/plasma polymer coatings and the large amount of suggested and tested approaches that were briefly summarized in this review, to date the use of such materials still remains very limited. This is partly due to the lack of clinical studies, as well as the not yet standardized methods applied for evaluating the antibacterial effects of produced coatings. The latter relates both to the choice of bacteria or procedures used for the quantification of antibacterial effects that makes it almost impossible to compare results reached by different groups. Because of this, there is a clear demand to propose/develop standardized, reliable, and high throughput validation methodologies for testing produced antibacterial coatings, which applies not only to the coatings produced by plasma-based methods but to other methods as well. In addition, all the experiments focused on determining the antibacterial effects of produced Ag/plasma polymer coatings were performed in vitro. It is well-known that under the more relevant in vivo conditions the performance of produced coatings may be largely altered, e.g., by the possible accumulation of dead bacteria or proteins on surfaces of produced coatings. Furthermore, other issues relate to factors which are often overlooked, such as wear resistance, long-term stability, the ability of Ag/plasma polymer nanocomposites to withstand common sterilization procedures, or, in the case of implanted materials, to integrate well with a host tissue. However, the use of plasma-based techniques—most likely in combination with other approaches—still presents itself as a vivid and auspicious option for the production of highly effective antibacterial coatings.

## 5. Conclusions

There has been enormous progress in silver/plasma polymer nanocomposite antibacterial coatings in the last two decades. As shown in this paper, numerous strategies have already been tested or are under consideration for the effective production of such materials. However, despite promising results, the field of Ag/plasma polymer antibacterial coatings still faces many challenges, meaning that better understanding and control of bactericide activity of the prepared coatings, as well as the development of new manufacturing procedures, are needed. 

## Figures and Tables

**Table 1:** Overview of the reported antibacterial effect of Ag/plasma polymer nanocomposites.

Deposition Method	Working Gas Precursor Target	Bacteria	Result	Reference
Simultaneous sputtering and plasma polymerization	Ag target Ar/DEGME	*Staphylococcus Epidermidis*	Inhibition zone 6 mm	[120]
Simultaneous sputtering and plasma polymerization	Ag target Ar/NH_3_/C_2_H_4_	*Pseudomonas aeruginosa,* *Staphylococcus aureus*	Bacterial growth down to 0%	[127]
Simultaneous sputtering and plasma polymerization	Ag target Ar/NH_3_/C_2_H_4_ Ar/ CO_2_/C_2_H_4_	*Pseudomonas aeruginosa,* *Staphylococcus aureus*	>7 log reduction	[128]
Simultaneous sputtering and plasma polymerization	Ag target Ar/HMDSO	*Escherichia coli,* *Staphylococcus aureus*	*E. Coli* > 6 log reduction *S. aureus* > 1 log	[129]
Simultaneous sputtering and plasma polymerization	Ag target Ar/HMDSO	*Saccharomyces cerevisiae*	1.9 log reduction	[130]
Simultaneous sputtering and plasma polymerization	Ag target polyaniline	*MRSA,* *MRSE, VRE,* *Escherichia coli* *Pseudomonas aeruginosa, * *Proteus mirabilis,* *Klebsiella pneumoniae*	Inhibition zone 20–22 mm for all tested bacteria. > 6 log reduction for *E. Coli* and MRSA	[131]
Simultaneous sputtering and plasma polymerization	Ag target C_2_H_2_	*Staphylococcus aureus*	99% of bacteria killed	[132]
Co-sputtering	Ag and PTFE targets	*Staphylococcus epidermidis, Staphylococcus aureus,* *Enterococcus faecalis,* *Pseudomonas aeruginosa,* *Escherichia coli,* *Klebsiella pneumoniae,* *Candida albicans*	7 log reduction for *S. aureus*	[126]
GAS system combined with plasma polymerization	Ag GAS Ar/n-hexane	*Escherichia coli*	Almost 3 log reduction	[133]
